# Is Adjuvant Chemotherapy Beneficial to All Patients With pT3N0M0 Stage Gastric Cancer?

**DOI:** 10.3389/fonc.2021.712432

**Published:** 2021-08-26

**Authors:** Jiao-Bao Huang, Jun Lu, Dong Wu, Bin-bin Xu, Zhen Xue, Guo-Sheng Lin, Hua-Long Zheng, Li-li Shen, Jia Lin, Jian-Wei Xie, Jia-Bin Wang, Jian-Xian Lin, Qi-Yue Chen, Long-Long Cao, Chao-Hui Zheng, Chang-Ming Huang, Ping Li

**Affiliations:** ^1^Department of Gastric Surgery, Fujian Medical University Union Hospital, Fuzhou, China; ^2^Key Laboratory of Ministry of Education of Gastrointestinal Cancer, Fujian Medical University, Fuzhou, China; ^3^Fujian Key Laboratory of Tumor Microbiology, Fujian Medical University, Fuzhou, China

**Keywords:** gastric cancer, pathological staging version, adjuvant chemotherapy, surgery, perineural invasion positive

## Abstract

**Background:**

The efficacy and benefits of adjuvant chemotherapy (AC) for patients with gastric cancer pT3N0M0 remain controversial.

**Methods:**

We prospectively collected and retrospectively analyzed 235 patients with pT3N0M0 gastric cancer who underwent radical resection between February 2010 and January 2016. Patients were divided into two groups: the surgery-alone (SA) group (n = 82) and the AC group (n = 153). We analyzed the effects of AC on the overall survival (OS) and recurrence-free survival (RFS), and the relationship between the number of chemotherapy cycles (CC) and recurrence rate (RR).

**Results:**

The 5-year OS and RFS of the participants were 80.9% and 87.7%, respectively, and those in the AC group were significantly higher than those in the SA group (86.9% *vs.* 69.5%, p = 0.003). The RFS of the AC and SA groups were 88.9% and 85.4%, respectively; the difference was not statistically significant (p = 0.35). The independent risk factors affecting the OS were perineural invasion-positive (PNI+) (HR = 2.64, 95%CI: 1.45–4.82, p = 0.003) and age ≥ 65 years (HR = 2.58, 95%CI: 1.39–4.8, p = 0.003). The independent risk factor affecting the RFS was also PNI+ (HR3.11; 95%CI: 1.48–6.54, p = 0.003). Stratified analysis revealed that postoperative AC can significantly improve the OS of PNI+ patients (AC group *versus* SA group: 84.1% *vs.* 45.5%, p = 0.001) and RFS (86.4% *vs.* 63.6%, p = 0.017). However, perineural invasion negative (PNI-) patients did not show the same results (p = 0.13 and p = 0.48, respectively). According to the number of CC, divided into CC < 3 groups and CC ≥ 3 groups, the cumulative RR in the CC ≥ 3 group of patients with PNI+ was significantly lower than that of the CC < 3 group (7.4% *vs.* 28.2%, p = 0.037).

**Conclusion:**

For pT3N0M0 gastric cancer patients with PNI+, at least three cycles of postoperative AC can significantly reduce the overall RR. This finding should be verified by using large external sample data.

## Introduction

Adjuvant chemotherapy (AC) for gastric cancer patients with postoperative pathological stages II and III has become widely accepted (1). However, the use of chemotherapy in patients with PT3N0M0 [stage IIA, according to the seventh edition of the American Joint Committee on Cancer (AJCC)] is still controversial in the guidelines of many Asian countries. This may be due to the exclusion of patients with pT3N0M0 stage in the Adjuvant Chemotherapy Trial of S-1 for Gastric Cancer (ATCS-GC) in Japan, which explored the standard treatment scheme for stages II and III gastric cancer after surgery. Therefore, there was no strong evidence of AC for patients in this stage (2). While postoperative AC was recommended for patients with postoperative stage IIA pT3N0M0 in the guidelines for the treatment of gastric cancer in Korea and China in 2018 and 2019, respectively (3, 4), it was still not recommended in the 2018 Japanese Guidelines for the Treatment of Gastric Cancer (5).

Lymph node metastasis is a high-risk factor for the recurrence of advanced gastric cancer (6). For pT2N1M0 patients who are also in stage IIA, the 2018 edition of the Japanese Guidelines recommends postoperative AC (5). Previous studies confirmed that invasion depth is a risk factor for the recurrence of advanced gastric cancer (7, 8). Therefore, exploring the prognostic factors of pT3N0M0 and the benefits of chemotherapy, and identifying subgroups that can benefit from AC will further help guide the clinical practice.

In this study, we retrospectively analyzed the prognostic risk factors and recurrence patterns of pT3N0M0 patients in our center over the last 6 years, and evaluated the effect of AC on the survival and recurrence in patients. To our knowledge, this is the first large-capacity center report to analyze the effect of postoperative AC on the recurrence pattern of pT3N0M0 gastric cancer patients.

## Methods

### Patient Characteristics and Clinical Data

**Figure 1:**
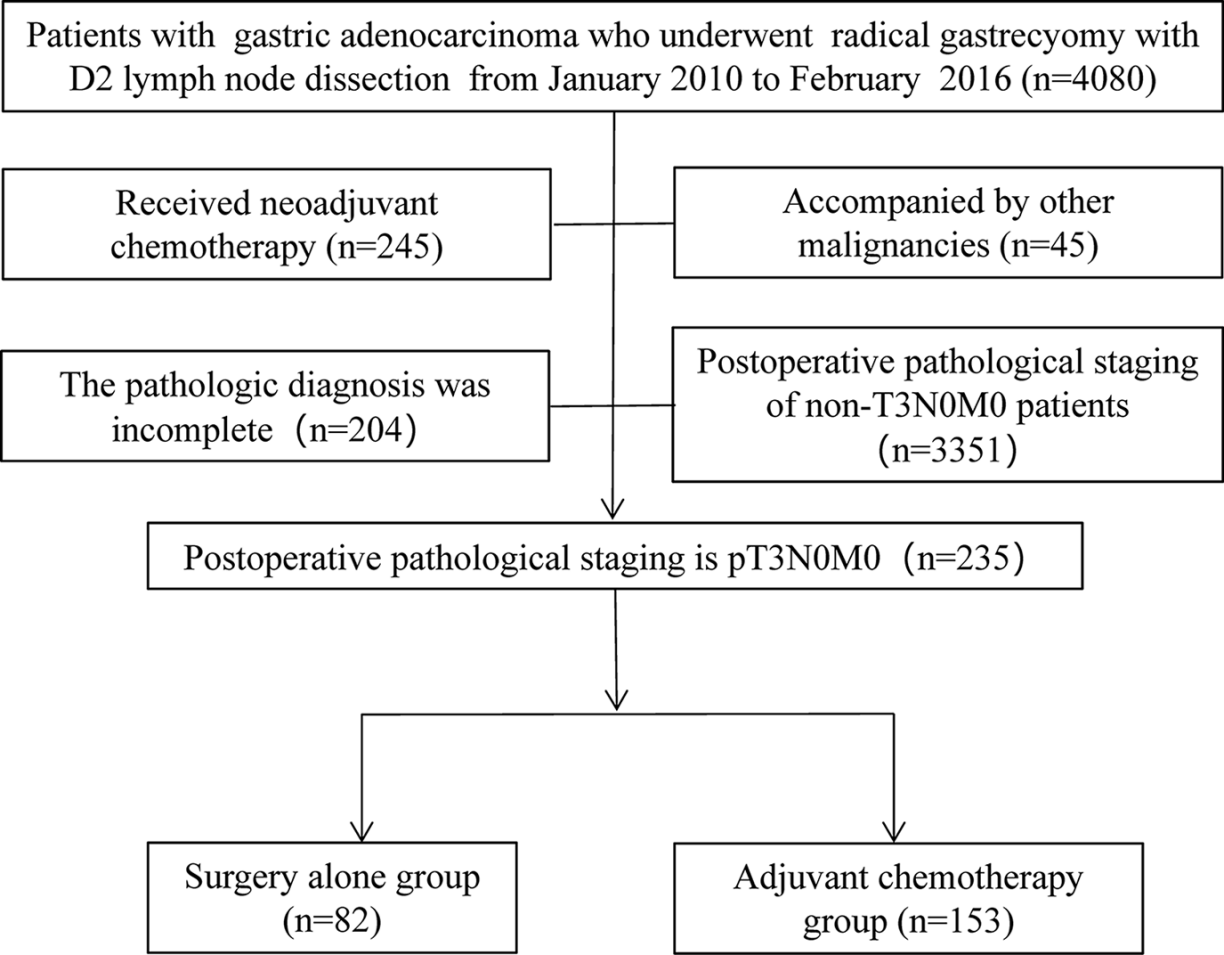
Study flowchart.

From February 2010 to January 2016, a total of 4,080 patients with gastric cancer underwent radical gastrectomy at Union Hospital, which is affiliated with Fujian Medical University. Of the 4,080 patients, 235 were diagnosed with pT3N0M0 (AJCC seventh edition) by pathological stage. All patients underwent gastrectomy according to different tumor locations, and patients who received neoadjuvant chemotherapy, synchronous tumors, or incomplete pathological diagnoses were excluded from the study (**Figure 1**). Depending on whether they received AC after surgery, patients were divided into two groups: surgery-alone (SA) and AC groups. The clinicopathological data of the patients are presented in **Table 1**. Perineural invasion was defined as hematoxylin-eosin (HE) staining in paraffin-embedded specimens of gastric cancer revealing the infiltration of tumor cells to the nerve bundle or perineurium.

**Table 1:** Comparison of the clinicopathological characteristics between adjuvant chemotherapy group and surgery alone group.

	Surgery alone n = 82	Adjuvant chemotherapy n = 153	P-Value
Age (%)			**<0.001**
≤65	38 (46.3)	114 (74.5)	
>65	44 (53.7)	39 (25.5)	
Gender (%)			**0.001**
Male	55 (67.1)	132 (86.3)	
Female	27 (32.9)	21 (13.7)	
ECOG (%)			**0.006**
0	14 (17)	25 (16.3)	
1	50 (61)	116 (75.8)	
2	18 (22)	12 (7.8)	
Tumor size (%)			0.842
<40 mm	52 (63.4)	95 (62.1)	
≥40 mm	30 (36.6)	58 (37.9)	
Tumor location (%)			0.633
Lower	30 (36.6)	49 (32.0)	
Middle	13 (15.9)	31 (20.3)	
Upper	28 (34.1)	58 (37.9)	
Mix	11 (13.4)	15 (9.8)	
Lymphatic invasion (%)			0.056
Positive	10 (12.2)	36 (23.5)	
Negative	72 (87.8)	117 (76.5)	
Perineural invasion (%)			0.872
Positive	22 (26.8)	44 (28.8)	
Negative	60 (73.2)	109 (71.2)	
Histologic type (%)			0.126
Differentiated	54 (65.9)	85 (55.6)	
Undifferentiated	28 (34.1)	68 (44.4)	
Complication (%)			0.751
Absent	77 (93.9)	142 (92.8)	
Present	5 (6.1)	11 (7.2)	

Bold characters indicate that the index has significant significance in the model (P < 0.05), and its significance is explained in the results section of the article.

### Chemotherapy Regimen

Since there is no consensus to date on the chemotherapy of patients with pathological stage pT3N0M0, decisions on chemotherapy and its regimens varies according to the preferences of the surgeons, oncologists, or patients. AC was generally carried out 3–4 weeks postoperatively, and the main regimen was either SOX or XELOX (79.1%). The SOX regimen was S-1 80 mg/m^2^/day, while the XELOX regimen with capecitabine (Xeloda^®^, Genentech, Inc., CA, USA) was 2,000 mg/m^2^/day, both for a total of 14 days, followed by 7 days of rest. This was followed by oxaliplatin 130 mg/m^2^/21 d for both groups and 3 weeks as a cycle (9). The number of chemotherapy cycles was determined by the tolerance and compliance of the patient, and the median chemotherapy cycle was 4 (range: 1–9). Therapeutic chemotherapy after recurrence was not included in the number of chemotherapy cycles.

### Follow-Up and Recurrence Pattern

The patients were followed up every 3–6 months within 2 years and every 6–12 months after 2 years. The follow-up included physical examination, laboratory examination (hematological indices, tumor markers), chest radiography, and total abdominal computed tomography (CT), and gastroscopy was completed annually. Recurrence-free survival (RFS) was defined as the interval between the date of operation and discovery of recurrence. Overall survival (OS) was defined as the interval between the date of operation and death or loss of follow-up for any cause.

Recurrence patterns are classified as local recurrence (including gastric stump cancer and local lymph node metastasis), peritoneal recurrence, and distant recurrence (liver, lung, bone, and distant lymph node metastasis). The detection of local and distant recurrence is usually confirmed by an abdominal enhanced CT or tissue biopsy. In our study, peritoneal recurrence was confirmed by as cites cytology or peritoneal nodules on CT scans. When multiple recurrence patterns were found simultaneously, they were displayed in the corresponding recurrence patterns.

### Statistical Analysis

The chi-square test was used to classify the variables in the clinical and pathological data, and Student’s t-test was used for continuous variables. OS and RFS curves were established using the Kaplan-Meier method and compared using the log-rank test. Cox regression hazard model was used to perform the analysis, and univariate and multivariate analyses were used to identify the risk factors for survival and recurrence, in which multivariate risk models were included when univariate p < 0.05, or had important clinical significance. The chi-square test was also used to compare different recurrence patterns, and the Fisher test was used when the sample size was less than five. The optimal number of chemotherapy cycles was intercepted by univariate regression of restricted cubic splines. R (https://www.r-project.org/) was used for statistical analysis.

## Results

### Clinicopathological Characteristics

Of the 235 patients with pT3N0M0 gastric cancer who underwent radical resection, 82 patients did not receive postoperative AC (SA group) and 153 patients received postoperative AC (AC group). **Table 1** shows the clinicopathological data of the two groups. Patients in the SA group were older (p < 0.001) and had a greater female-to-male ratio (p = 0.001). There were no significant differences in other pathological data between the two groups.

### Benefits of AC on OS and RFS in All Patients

**Figure 2:**
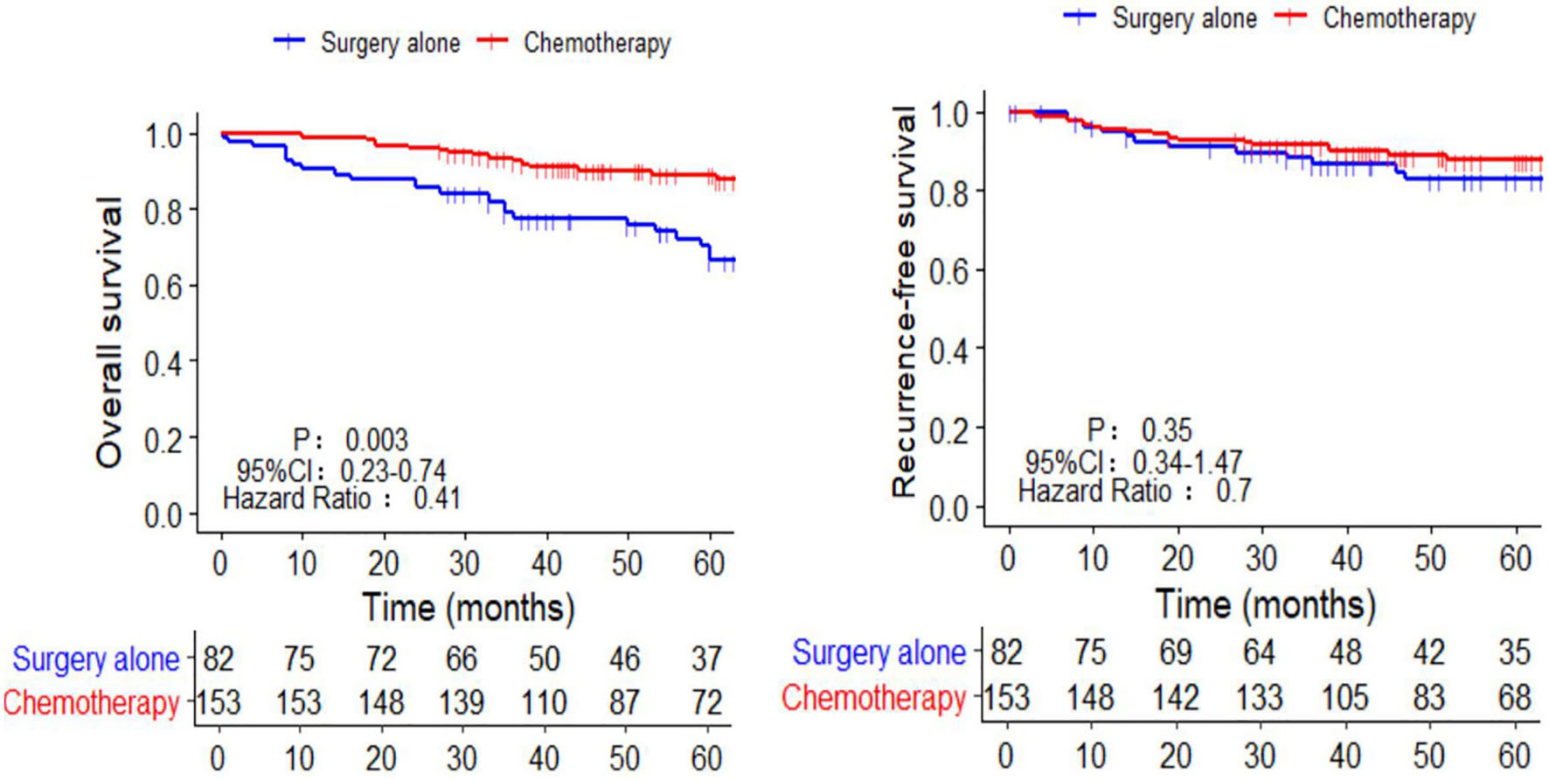
Kaplan-Meier survival curves of chemotherapy and surgery alone in the OS and RFS of pT3N0M0 patients. OS, overall survival; RFS, recurrence-free survival.

The 5-year OS in the AC group was significantly higher than that in the SA group (86.9% *vs.* 69.5%, respectively; p = 0.003). However, the 5-year RFS rates were similar (88.9% *vs.* 85.4%, respectively; p = 0.35) (**Figure 2**). The independent risk factors for survival were age ≥ 65 years (HR = 2.58, 95%CI: 1.39–4.8, p = 0.003) and perineural invasion positive (PNI+) (HR = 2.64, 95%CI: 1.45–4.82, p = 0.003). The effect of AC on survival was also significant. (HR = 0.46, 95%CI: 0.25–0.86, p = 0.019) (**Table 2**). PNI+ was the only independent risk factor for recurrence (HR = 3.11, 95%CI: 1.48–6.54, p = 0.003) (**Table 3**).

**Table 2:** Univariate and multivariate analyses of overall survival.

	Univariate analysis			Multivariate analysis		
	HR	95%CI	P-Value	HR	95%CI	P-Value
Age (y)						
<65						
≥65	3.03	1.81–6.02	<0.001	**2.58**	**1.39**–**4.80**	**0.003**
Gender						
Male						
Female	0.69	0.4–3.04	0.691			
ECOG						
0						
1	0.56	0.78–2.13	0.351			
2	1.83	0.45–1.86	0.569			
Adjuvant treatment						
Surgery alone						
Chemotherapy	0.41	0.23–0.74	0.003	**0.46**	**0.25**–**0.86**	**0.019**
Lymphatic invasion						
Absent						
Present	1.30	0.66–2.56	0.451			
Perineural invasion						
Absent						
Present	2.61	1.44–4.74	0.002	**2.64**	**1.45**–**4.82**	**0.003**
Tumor location						
Low						
Middle	1.12	0.47–2.66	0.791			
High	1.09	0.54–2.20	0.817			
Mix	1.28	0.49–3.30	0.615			
Tumor size(mm)						
<40						
≥40	1.77	0.99–3.18	0.056			
Histologic type						
Differentiated						
Undifferentiated	0.78	0.42–1.44	0.429			
Complication						
Absent						
Present	1.60	0.63–4.05	0.326			

Bold characters indicate that the index has significant significance in the model (P < 0.05), and its significance is explained in the results section of the article.

**Table 3:** Univariate and multivariate analyses of recurrence-free survival.

	Univariate analysis			Multivariate analysis		
	HR	95%CI	P-Value	HR	95%CI	P-Value
Age (y)						
<65						
≥65	1.659	0.80–3.45	0.176			
Gender						
Male						
Female	0.79	0.3–2.06	0.327			
ECOG						
0						
1	0.76	0.25–2.30	0.631			
2	2.95	0.93–9.41	0.068			
Adjuvant treatment						
Surgery alone						
Chemotherapy	0.7	0.34–1.47	0.352	0.56	0.26–1.20	0.138
Lymphatic invasion						
Absent						
Present	1.89	0.86–4.15	0.113	1.86	0.83–4.16	0.341
Perineural invasion						
Absent						
Present	3.08	1.48–6.41	**0.004**	**3.11**	**1.48**–**6.54**	**0.003**
Tumor location						
Low						
Middle	1.69	0.61–4.66	0.313			
High	1.06	0.41–2.76	0.899			
Mix	1.89	0.62–5.77	0.265			
Tumor size (mm)						
<40						
≥40	1.64	0.79–3.40	0.183	1.68	0.81–3.49	0.164
Histologic type	1.21	0.77–1.91	0.403			
Differentiated						
Undifferentiated	1.36	0.66–2.82	0.408			
Complication						
Absent						
Present	1.56	0.47–5.16	0.465			

RFS, recurrence-free survival. Bold characters indicate that the index has significant significance in the model (P < 0.05), and its significance is explained in the results section of the article.

### Benefits of AC on OS and RFS in Patients With PNI+

**Figure 3:**
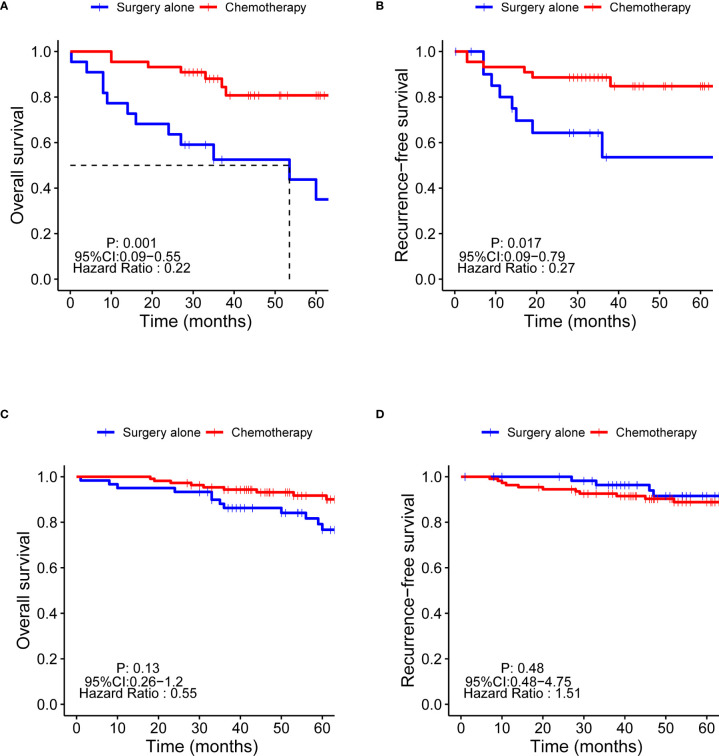
Kaplan-Meier survival curves of the OS and RFS of chemotherapy in patients with PNI+ and PNI-. OS, overall survival; RFS, recurrence-free survival; PNI+, perineural invasion-positive; PNI-, perineural invasion-negative. **(A)** OS of PNI+ patients; **(B)** RFS of PNI+ patients; **(C)** OS of PNI- patients; **(D)** RFS of PNI+ patients.

The OS and RFS of patients with PNI+ 5 years post-procedure were significantly lower than those of patients with PNI- (OS: 71.2% *vs.* 84.6%, respectively; p = 0.002; RFS: 78.8% *vs.* 91.1%, respectively; p = 0.003) (**Supplementary Figure 1**). Patients with PNI+ 5 years post-procedure were divided into two groups according to chemotherapy and non-chemotherapy; OS (84.1% *vs*. 45.5%, respectively; p = 0.001) and RFS (86.4% *vs.* 63.6%, respectively; p = 0.017) were significantly higher in the chemotherapy group (**Figures 3A, B**). However, there was no significant difference in the OS and RFS between the chemotherapy and non-chemotherapy groups in patients with PNI- (**Figures 3C, D**).

### Effect of AC on the Recurrence Pattern of All Patients

The overall recurrence rate of the whole group was 12.3% (29/235), 11.1% (17/153) in the AC group, and 14.6% (12/82) in the SA group. There was no significant difference between the two groups (p = 0.331). The local recurrence rate was 4.7% (11/235) in all participants, 5.2% (8/153) in the AC group, and 3.7% (3/82) in the SA group. The recurrence rate of the peritoneum was 3% (7/235) in all patients, 2% (3/153) in the AC group, and 4.9% (4/82) in the SA group. The distant recurrence rates were 6.8% (16/235) for all patients, 5.9% (9/153) in the AC group, and 8.5% (7/82) in the SA group. However, there was no significant difference in the recurrence of each site after chemotherapy (p = 0.751, p = 0.698, and p = 0.765, respectively) (**Supplementary Figure 2**).

In terms of the recurrence time, the recurrence rate within 1 year was 3.8% (9/235) for all patients, 3.9% (6/153) in the AC group, and 3.7% (3/82) in the SA group. The current rate at 1–2 years was 3.8% (9/235) for all patients, 3.3% (5/153) in the AC group, and 4.9% (4/82) in the SA group. The recurrence rate after 2 years was 4.7% (11/235) for all patients, 3.9% (6/153) in the AC group, and 6.1% (5/82) in the SA group. There was no significant difference in the recurrence time between the two groups (p = 0.727, p = 0.756, and p = 0.834, respectively) (**Supplementary Figure 2**).

### The Influence of PNI+ on the Recurrence Pattern of Patients

The total and peritoneal recurrence rates of patients with PNI+ were 21.2% (14/66) and 7.6% (5/66), respectively, while those of patients with PNI- were 8.9% (15/169) and 1.2% (2/169), respectively. This difference was statistically significant (p = 0.01, p = 0.02). The local and distant recurrence rates were 7.6% (5/66) and 9.1% (6/66), respectively. However, the proportion of patients with PNI was 3.6% (6/169) and 5.9% (10/169), with no significant difference (p = 0.19, p = 0.39) (**Supplementary Figure 3**).

The recurrence rate within 1 year was 9.1% (6/66) in patients with PNI+ and 1.8% (3/169) in patients with PNI- (p = 0.013), and the 1 to 2-year recurrence rate was 9.1% in patients with PNI+ and 1.8% in patients with PNI- (p = 0.013). The recurrence rate after 2 years in patients with PNI+ was 3% (2/66), and that in patients with PNI- was 5.3% (9/169) (p = 0.735) (**Supplementary Figure 3**).

### The Influence of Chemotherapy Cycles on the Recurrence Rate of Patients With PNI+

**Figure 4:**
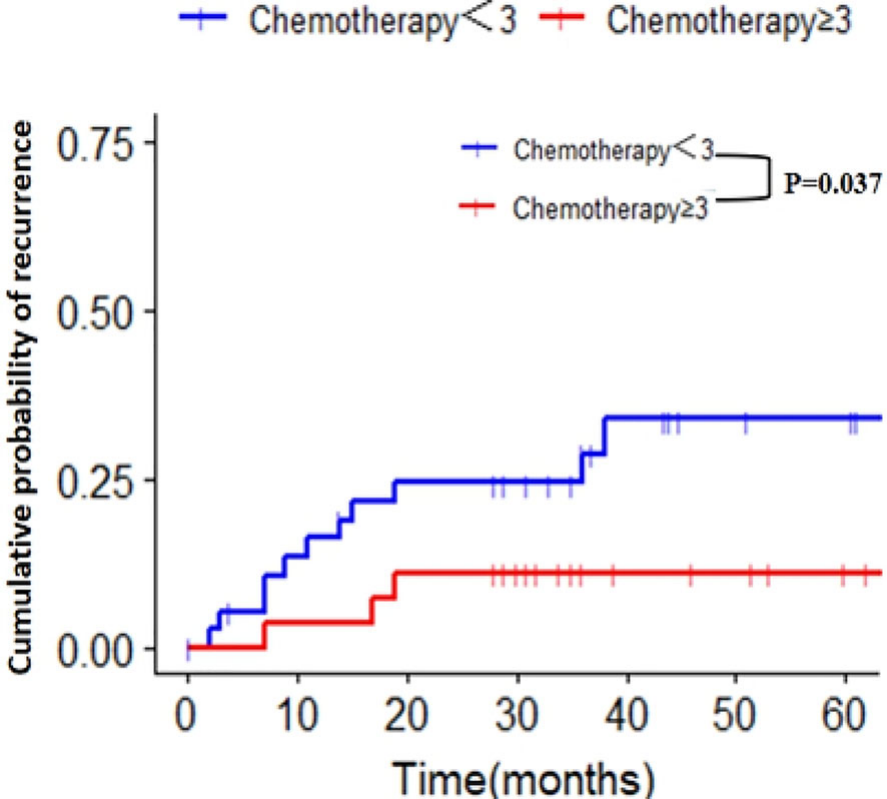
Cumulative recurrence rate of two groups with different chemotherapy cycles in patients with PNI+. PNI+, perineural invasion-positive.

To explore the influence of the chemotherapy cycle on the recurrence of patients with PNI+, we analyzed the relationship between the cumulative recurrence rate and chemotherapy cycle by univariate regression of restricted cubic spline, and found that three cycles of the postoperative chemotherapy were the best cut-off values (**Supplementary Figure 4**). Patients with PNI+ were divided into two groups: chemotherapy period < 3 and chemotherapy period ≥ 3. The 5-year cumulative recurrence rates of the two groups were as follows: chemotherapy cycle < 3 group, 28.2% (11/39); chemotherapy cycle ≥ 3 group, 7.4% (2/27); the difference between the two groups was statistically significant (p = 0.037) (**Figure 4**). The cumulative recurrence rates of the peritoneum were 12.8% (5/39) and 7.4% (2/27), respectively, with no significant difference between the two groups (p = 0.691) (**Supplementary Figure 5**).

## Discussion

A 2010 meta-analysis reported the difference in the prognosis between AC and SA after gastric cancer surgery in the last 40 years (1970–2009). Postoperatively, AC was found to be significantly better than SA in the OS and RFS, and the 5-year survival rate after AC increased from 49.6% to 55.3% (10). However, the analysis did not explore the benefits of AC at different stages. Subsequently, two large randomized clinical trials (capecitabine and oxaliplatin adjuvant study in stomach cancer (11) and ATCS-GC) compared the benefits of AC with SA in stages II and III gastric cancer. The results also showed that patients with gastric cancer could benefit from AC (2, 12). Although AC has been recommended for pT3N0M0 in stage IIA in the guidelines of China and South Korea, recently published Japanese guidelines for gastric cancer have still not recommended the use of AC for pT3N0M0 (5). The reason may be that the update of the pathological staging version leads to a lack of evidence of chemotherapy in the special pathological staging of “invading the subserous”. In the ATCS-GC and CLASSIC studies, “invading the subserous” was defined as T2b [referring to the Japan Gastric Cancer Treatment Protocol version 13 (JGCTP^13th^) and AJCC^6th^], while the pT2bN0M0 pathological stage was IB stage. Therefore, this group of patients could not enter the trial group as stage II. However, “invading the subserous” has been defined as T3 (see **Table 1**) in the JGCTP^14th^ and the AJCC^7th^. Therefore, the benefit of AC in patients with gastric cancer with subserosa invasion and no lymph node metastasis is still controversial and needs to be further explored.

Recently, a large-volume study by Kang et al. compared the difference in the prognosis between AC and SA in patients with pT3N0M0, and concluded that AC did not show oncological benefits (13). In contrast to that study, the patients included in our study were all treated after 2010, and according to AJCC^7th^, “invasion of serosa” is defined as T3 (patients of Kang from 2000 to 2018), which can reduce the stage bias caused by the update of the pathological stage version. Here, we also performed a further stratified analysis of pT3N0M0 and found that PNI+ is an independent risk factor for pT3N0M0 patients, and AC can significantly reduce the recurrence rate of patients with PNI+ in pT3N0M0.

Previously, Jiang et al. and Aurello et al. reported that the positive rate of perineural invasion in gastric cancer was 35.9% and 45.6%, respectively (14, 15), while in the current study, the positive rate of perineural invasion was 26.1%. The reason for the low incidence is that this study was only aimed at patients with pT3N0M0. Although it has been found that the poor prognosis of gastric cancer patients with PNI+ (16) and the incidence of peritoneal recurrence is higher (17), to our knowledge, this is the first report to describe the relationship between pT3N0M0 and patients with PNI+. The mechanism underlying the effect of PNI+ on the recurrence of gastric cancer remains unclear. Previous studies have suggested that gastric cancer cells invading the nerve will spread along the nerve space, resulting in an early recurrence and a poor prognosis with reports of cancer-related pain and digestive juice secretion disorder (18). Based on this principle, some experts have further proposed that cancer cells remaining in the extragastric nerve after gastrectomy may move into the peritoneal cavity and enter the peritoneum (17), causing peritoneal spread or metastasis. Suzuki et al. found that patients who received AC exhibited a better RFS and OS than those who did not receive AC among patients with stage III colorectal cancer and PNI+ (19). The role of chemotherapeutic drugs in patients with PNI+ is mainly related to the “perineural niche.” Nerve cells have a nutrient-rich perineural space, are characterized by extensive vascular and lymphatic supply, and can easily reach the tumor site, which makes patients with PNI+ more sensitive to chemotherapeutic drugs (20).

At present, there is no clear definition of the optimal cycle number of postoperative AC for gastric cancer; in a clinical trial of ATCS-GC (12), the included patients received eight cycles of S-1 regimen. Yamada et al. compared the efficacy of CS (cisplatin +S-1) and SOX regimen, with median chemotherapy cycles of five and seven, respectively (21). In our retrospective study, the median number of chemotherapy cycles for patients with PNI+ was 3 (range: 1–8), and 86.3% of the patients received the SOX regimen. We analyzed the relationship between the recurrence rate and the number of chemotherapy cycles by univariate risk regression of restricted cubic splines and found that three cycles were the best cut-off points. When patients were divided based on the number of chemotherapy cycles (<3 and ≥3 groups), the cumulative recurrence rate of the <3 group was significantly higher than that of the ≥3 group (28.2% *vs.* 7.4%, p = 0.037). In addition, the peritoneal recurrence rate in the chemotherapy cycle <3 group was also higher than that in the ≥3 cycle group (12.8% *vs.* 7.4%), although the difference was not statistically significant, which may be due to the small number of cases in the subgroup. Therefore, the above results suggest that for patients with pT3N0M0 who were diagnosed relatively early, at least three cycles of AC should be administered to effectively reduce postoperative recurrence.

The limitations of this study are as follows: 1) for the retrospective study, patients failed to receive standardized treatment after surgery (including chemotherapeutic drugs and the number of chemotherapy cycles) and 2) due to the incomplete data of chemotherapy toxicity recorded, the toxicity of different cycles of chemotherapy could not be compared. In addition, because this study was conducted at a single center, the sample size was small. However, to our knowledge, this study is the first to find that PNI+ is related to the prognosis of patients with pT3N0M0 gastric cancer; thus, large-volume random clinical data are needed to verify the results.

## Conclusion

This study provides clinical evidence for the benefit of postoperative AC for patients with gastric cancer in the eighth edition of AJCC staging for pT3N0M0. Our study revealed for the first time that PNI+ is an independent risk factor for the prognosis of patients with pT3N0M0 gastric cancer. For patients with pT3N0M0 gastric cancer with PNI+, postoperative AC for at least three cycles can significantly reduce the overall recurrence rate. This finding needs to be verified by using large external sample data.

## Data Availability Statement

The raw data supporting the conclusions of this article will be made available by the authors, without undue reservation.

## Ethics Statement

The studies involving human participants were reviewed and approved by Ethics Committee of Union Medical College Hospital Affiliated to Fujian Medical University. Written informed consent for participation was not required for this study in accordance with the national legislation and the institutional requirements.

## Author Contributions

Study concepts: PL, CH, JuL, and JH. Study design: PL, CH, JuL, and JH. Data acquisition: All authors. Quality control of data and algorithms: PL and CH. Data analysis and interpretation: JH, JuL, DW, and PLi. Statistical analysis: JH, JuL, DW, and BX. Manuscript preparation: JH, JuL, PL, and CH. Manuscript editing: JH, JuL, PL, and CH. Manuscript review: All authors. All authors contributed to the article and approved the submitted version.

## Funding

This work was supported by the Joint Funds for the Innovation of Science and Technology, Fujian Province (No.2017Y9011, No.2017Y9004, and No. 2019Y9098).

## Conflict of Interest

The authors declare that the research was conducted in the absence of any commercial or financial relationships that could be construed as a potential conflict of interest.

## Publisher’s Note

All claims expressed in this article are solely those of the authors and do not necessarily represent those of their affiliated organizations, or those of the publisher, the editors and the reviewers. Any product that may be evaluated in this article, or claim that may be made by its manufacturer, is not guaranteed or endorsed by the publisher.

## Supplementary Material

Supplementary Figure 1 Kaplan-Meier survival curves of OS and RFS in PNI+ and PNI-. OS, overall survival; RFS: recurrence-free survival; PNI+, perineural invasion-positive; PNI-, perineural invasion-negative.

Supplementary Figure 2 Recurrence pattern and recurrence time in the adjuvant chemotherapy and surgery alone groups.

Supplementary Figure 3 Recurrence pattern and time of PNI+ and PNI-. PNI+, perineural invasion-positive; PNI-, perineural invasion-negative.

Supplementary Figure 4 Scatter plot of log HR by chemotherapy cycle with RCS fit. HR, hazard ratio; RCS, recurrence-free survival.

Supplementary Figure 5 Cumulative peritoneal recurrence rate of two groups with different chemotherapy cycles in patients with PNI+. PNI+, perineural invasion-positive.
