# Supplementary figures and images for: Is Adjuvant Chemotherapy Beneficial to All Patients With pT3N0M0 Stage Gastric Cancer?
Source: Front Oncol. 2021 Aug 26;11:712432. doi: 10.3389/fonc.2021.712432 (PMC8428976; doi:10.3389/fonc.2021.712432)

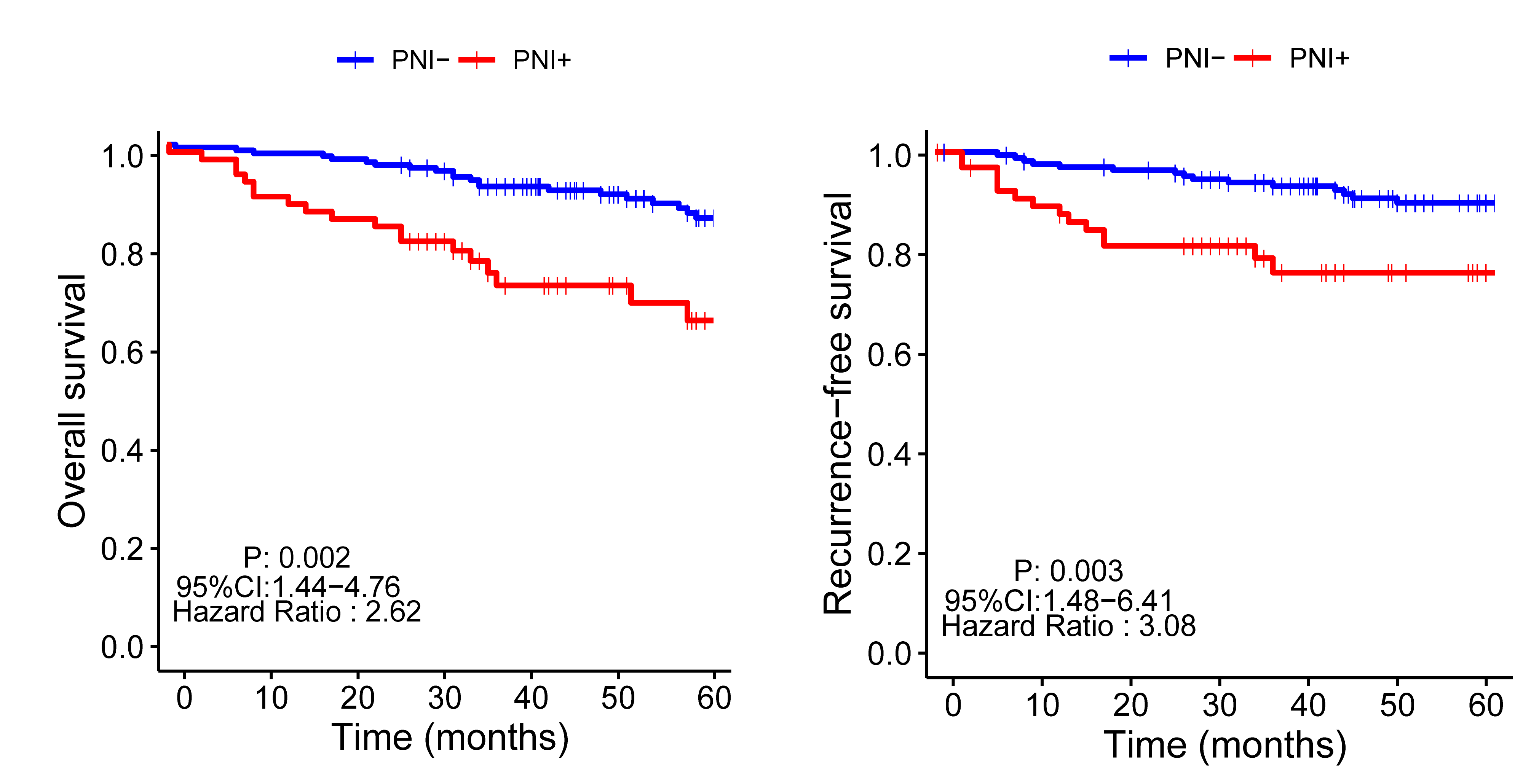

Supplement: Supplementary Figure 1 — Kaplan-Meier survival curves of OS and RFS in PNI+ and PNI-. OS, overall survival; RFS: recurrence-free survival; PNI+, perineural invasion-positive; PNI-, perineural invasion-negative. [file Image_1.tif]

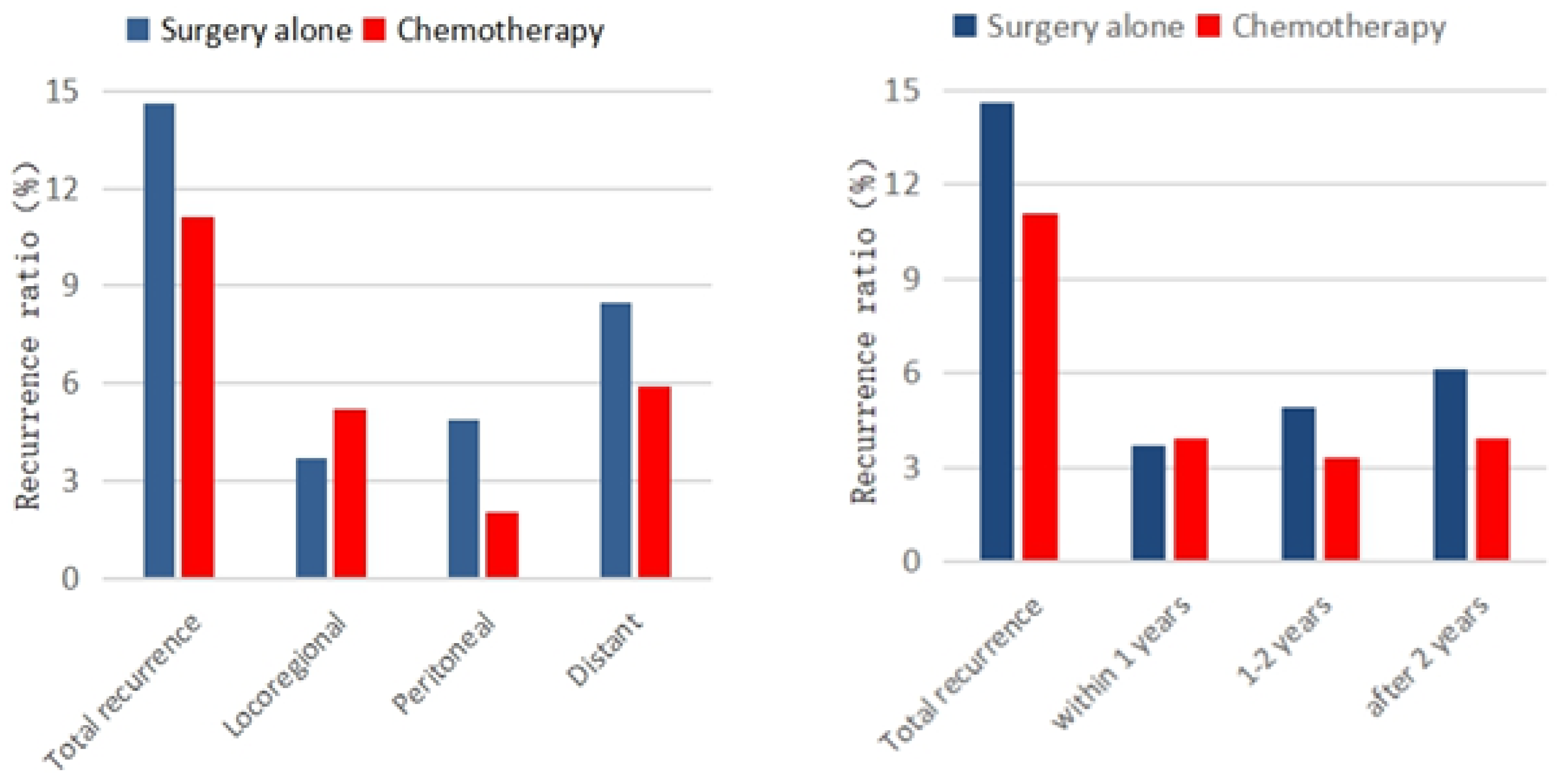

Supplement: Supplementary Figure 2 — Recurrence pattern and recurrence time in the adjuvant chemotherapy and surgery alone groups. [file Image_2.tif]

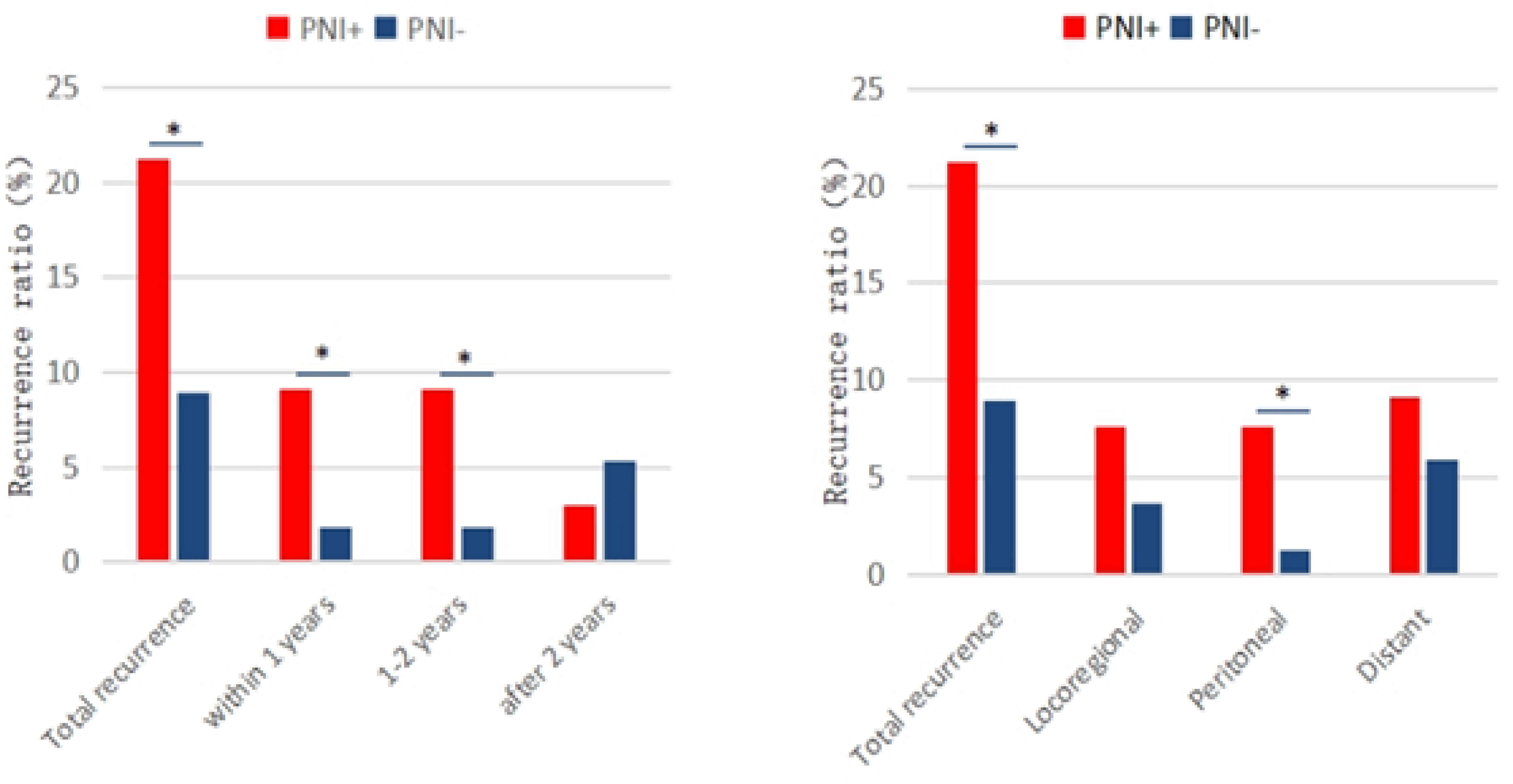

Supplement: Supplementary Figure 3 — Recurrence pattern and time of PNI+ and PNI-. PNI+, perineural invasion-positive; PNI-, perineural invasion-negative. [file Image_3.tif]

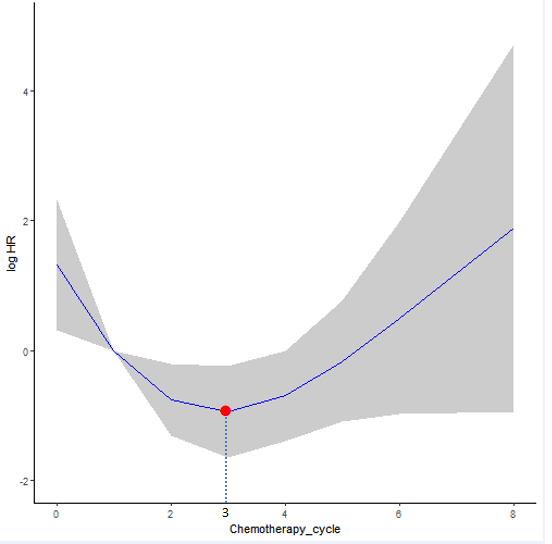

Supplement: Supplementary Figure 4 — Scatter plot of log HR by chemotherapy cycle with RCS fit. HR, hazard ratio; RCS, recurrence-free survival. [file Image_4.tif]

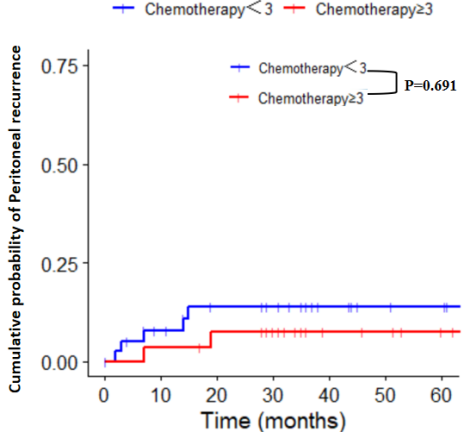

Supplement: Supplementary Figure 5 — Cumulative peritoneal recurrence rate of two groups with different chemotherapy cycles in patients with PNI+. PNI+, perineural invasion-positive. [file Image_5.tif]
