# Supplementary material for: Is Adjuvant Chemotherapy Beneficial to All Patients With pT3N0M0 Stage Gastric Cancer?
Source: Front Oncol. 2021 Aug 26;11:712432. doi: 10.3389/fonc.2021.712432 (PMC8428976; doi:10.3389/fonc.2021.712432)
Supplement: Supplementary file 6 [file Table_1.doc]

**eTable** 1 Changes to the pathological staging version

|  | JGCTP(13^th^)  AJCC（6^th^） |  | JGCTP(14^th^)    AJCC（7^th^） |
| --- | --- | --- | --- |
| Tumor invades  the subserosa | T2b |  | T3 |
| Pathological staging without lymph node metastasis | IB |  | IIA |

JGCTP Japan Gastric Cancer Treatment Protocol

AJCC American Joint Committee on Cancer

**eTable 2** The relationship between chemotherapy cycle（CC） and age and ECOR score

|  | CC＜3  n=143 | CC≥3  n=92 | P value |
| --- | --- | --- | --- |
| Age（%） |  |  | **＜0.001** |
| ≤65 | 78（54.5） | 74（80.4） |  |
| ＞65 | 65（45.5） | 18（19.6） |  |
| ECOG（%） |  |  | **0.04** |
| 0 | 24（16.8） | 15（16.3） |  |
| 1 | 93（65） | 73（79.3） |  |
| 2 | 26（18.2） | 4（4.3） |  |
